# Human visceral and subcutaneous adipose stem and progenitor cells retain depot-specific adipogenic properties during obesity

**DOI:** 10.3389/fcell.2022.983899

**Published:** 2022-10-17

**Authors:** Neha Mathur, Mai C. K. Severinsen, Mette E. Jensen, Lars Naver, Maren Schrölkamp, Matthew J. Laye, Matthew J. Watt, Søren Nielsen, Rikke Krogh-Madsen, Bente Klarlund Pedersen, Camilla Scheele

**Affiliations:** ^1^ The Centre for Physical Activity Research, Department of Infectious Diseases and CMRC, Rigshospitalet, Faculty of Health Sciences, University of Copenhagen, Copenhagen, Denmark; ^2^ Novo Nordisk Foundation Center for Basic Metabolic Research, University of Copenhagen, Copenhagen, Denmark; ^3^ Department of Gastroenterology, Hvidovre Hospital, Hvidovre, Denmark; ^4^ Department of Anatomy and Physiology, University of Melbourne, Melbourne, VIC, Australia

**Keywords:** human adipocytes, adipogenesis, immunogenic adipocytes, obesity, visceral adipocytes, subcutaneous adipocytes

## Abstract

Abdominal obesity associates with cardiometabolic disease and an accumulation of lipids in the visceral adipose depot, whereas lipid accumulation in the subcutaneous depot is more benign. We aimed to further investigate whether the adipogenic properties where cell-intrinsic, or dependent on a depot-specific or obesity-produced microenvironment. We obtained visceral and subcutaneous biopsies from non-obese women (*n* = 14) or women living with morbid obesity (*n* = 14) and isolated adipose stem and progenitor cells (ASPCs) from the stromal vascular fraction of non-obese (*n* = 13) and obese (*n* = 13). Following *in vitro* differentiation into mature adipocytes, we observed a contrasting pattern with a lower gene expression of adipogenic markers and a higher gene expression of immunogenic markers in the visceral compared to the subcutaneous adipocytes. We identified the immunogenic factor *BST2* as a marker for visceral ASPCs. The effect of obesity and insulin resistance on adipogenic and immunogenic markers in the *in vitro* differentiated cells was minor. In contrast, differentiation with exogenous Tumor necrosis factor resulted in increased immunogenic signatures, including increased expression of *BST2*, and decreased adipogenic signatures in cells from both depots. Our data, from 26 women, underscore the intrinsic differences between human visceral and subcutaneous adipose stem and progenitor cells, suggest that dysregulation of adipocytes in obesity mainly occurs at a post-progenitor stage, and highlight an inflammatory microenvironment as a major constraint of human adipogenesis.

## Introduction

The regional distribution of adipose tissue exerts a profound influence on systemic metabolism and confers an altered risk for the development of metabolic diseases (Lee et al., 2013). Abdominal obesity, which is characterized by increased lipid accumulation in visceral adipose tissue (VAT), is associated with the development of cardiovascular disease, type 2 diabetes and all-cause mortality (Yusuf et al., 2005; Pischon et al., 2008). In contrast, accumulation of subcutaneous adipose tissue (SAT) is associated with beneficial metabolic properties (Manolopoulos et al., 2010) and is linked to reduced incidence of metabolic disease (Snijder et al., 2004; Jensen, 2008; Karpe and Pinnick, 2014). VAT is more lipolytic than SAT (Östman et al., 1979). Thus, VAT seem to function more as a short-term storage of lipids, whereas SAT has a higher lipid storage capacity and display a decreased lipolysis already in an overweight state (Spalding et al., 2017) . Differences in lipid storing capacity seems to be preserved in isolated adipose stem and progenitor cells (ASPCs) as ASPCs derived from VAT demonstrated a lower adipogenic capacity compared to ASPCs derived from SAT (Baglioni et al., 2012). In rodents, human VAT is probably best modelled by the perigonadal adipose depot. This adipose depot is well studied and is characterized by resident immune cells and by its expansion in obesity (Vishvanath and Gupta, 2019). Rodent SAT appears in the inguinal adipose depot. This depot respond to the microenvironment by switching between a thermogenic and metabolically healthy state in young mice, and when subjected to cold, and a fibrotic and inflamed state during aging (Horvath and Scheele, 2022; Wang et al., 2022). Recent single cell and single nuclei studies have provided additional insights to several different types of ASPCs and adipocytes with specialized functions (Min et al., 2019; Raajendiran et al., 2019; Vijay et al., 2019; Bäckdahl et al., 2021; Emont et al., 2022). Interestingly, these studies have also revealed previously unknown differences between adipose depots in mice and humans. For example, Emont and co-authors found that, in mice, the cell-type abundance in white adipose tissue was highly dependent on body weight, with only minor variation between depots. In contrast, humans displayed differences in a subset of adipocyte types between visceral and subcutaneous depots, with a lipogenic subtype in the subcutaneous depot and a thermogenic subtype in the visceral depot (Emont et al., 2022; Scheele and Nielsen, 2022). This emphasize a species difference in adipose tissue depots as browning of white adipose tissue in rodents mainly occur in the inguinal subcutaneous adipose depot (Seale et al., 2011). Importantly, other adipose-resident cell types will also contribute to the phenotype, including macrophages which accumulate during obesity and obesity-induced insulin resistance (Bai and Sun, 2015) and associate with low-grade inflammation (Weisberg et al., 2003; Xu et al., 2003). However, recent comparisons at single cell level reported only minor differences in immune cell populations between VAT and SAT (Emont et al., 2022). Thus, the interplay between VAT and SAT intrinsic adipogenic properties and an inflammatory microenvironment remains elusive. In the current study, we aimed to explore the differences between adipose stem and progenitor cells (ASPCs) derived from VAT and SAT of obese and non-obese women, with a specific focus on their intrinsic and extrinsic adipogenic properties and immunogenic signatures.

## Materials and methods

### Subjects

Patients undergoing laparoscopic cholecystectomy or Roux-en-Y gastric bypass operation at the department of Gastroenterology, Hvidovre Hospital, University of Copenhagen, Denmark, were asked to participate in the study. Women that were either non-obese (*n* = 14; BMI<30 kg/m^2^) or living with obesity (*n* = 14; BMI<35 kg/m^2^) were included in the study (Table 1). The subjects provided written and informed consent, and the study protocol was approved by the Scientific Ethics Committee of the Capital Region of Denmark, journal number H-A-2008-081 and performed in accordance with the Helsinki declaration. The WHO criteria for glucose tolerance was applied (WHO, 2006). The subjects completed a two-hour oral glucose tolerance test (OGTT) before the operation, except for five cholecystectomy patients (*n* = 5), who, due to logistical reasons, underwent the OGTT four to 8 weeks post-surgery after a full recovery. Briefly, after an overnight fast, patients underwent a two-hour OGTT (75g glucose dissolved in 350 ml water). Blood samples for measurement of plasma levels of glucose and insulin were collected before and after the 120 min. Insulin resistance (IR) was estimated using the Homeostasis Model Assessment (HOMA)-IR (((glucose (mmol/L) × insulin (μmol/ml))/22.5 (Matthews et al., 1985)) from fasting venous blood samples. As standard procedure prior to Roux-en-Y gastric bypass operation at Hvidovre hospital, patients were advised to lose 8% of their body weight 3 months prior to their operation, while such criteria was not a requirement for cholecystectomy patients.

**TABLE 1:** Subject characteristics of normal glucose tolerant (NGT) non-obese women (BMI<30 kg/m^2^) and women living with obesity (BMI>35 kg/m^2^) undergoing laparoscopic cholecystectomy or gastric by-pass operation. Data are mean ± SEM (standard error of the mean). BMI, Body Mass Index; HDL, High Density Lipid; HOMA-IR, Homeostasis Model Assessment Insulin Resistance; p, plasma; LDL, Low Density Lipid. The two groups were compared using unpaired t-tests, *p* < 0.5 was considered significant. Clinical data was unavailable for some of the subjects. For three subjects from the group with obesity, some clinical data were missing including p-insulin, 120 min p-glucose, HOMA-IR and BMI. For one subject with obesity, p-HDL, p-LDL, p-triglyceride and fasting p-glucose data were missing. For one non-obese subject fasting p-glucose and HOMA-IR data were missing.

Subject characteristics			
NGT women	Non-Obese	Obese	p value
n	14	14	
BMI ± SEM (kg/m^2^)	24.93 ± 0.88	41.12 ± 1.05	<0.0001
Age ± SEM	45.57 ± 3.42	36.36 ± 2.21	<0.05
HOMA-IR* ± SEM	1.65 ± 0.25	3.46 ± 0.63	<0.05
Fasting p-Glucose (mmol/L)	4.85 ± 0.12	5.63 ± 0.14	<0.001
120 min p-Glucose (mmol/L)	5.90 ± 0.27	6.00 ± 0.22	NS
Fasting Insulin (pmol/L)	47.91 ± 6.55	79.36 ± 13.36	<0.05
p-Triglyceride (mmol/L)	1.03 ± 0.13	1.43 ± 0.15	0.05
p-HDL (mmol/L)	1.44 ± 0.10	1.08 ± 0.05	<0.01
p-LDL (mmol/L)	2.76 ± 0.18	3.53 ± 0.20	<0.01

### Adipose tissue biopsies

Abdominal subcutaneous adipose tissue biopsies were collected at the start of either the laparoscopic cholecystectomy or the Roux-en-Y gastric bypass operation from the surgical incision cut near the umbilicus. Visceral adipose tissue biopsies were obtained from the greater omentum. Biopsies were collected from planned incisions and surgically removed by the relevant surgeon. Biopsies were immediately rinsed with saline to wash off the blood, and any visible vasculature was removed. Subsequently biopsies from non-obese (*n* = 14) and obese (*n* = 14) subjects was flash frozen in liquid nitrogen and stored at −80°C for RNA purification. For *n* = 13 of the non-obese and *n* = 13 of the obese subjects, half of the biopsy was placed in DMEM/F12 for isolation of ASPCs.

### Isolation of human adipose stem and progenitor cells

ASPCs were isolated from the non-obese (*n* = 13) and obese (*n* = 13) group. Adipose biopsies were minced with scissors to small pieces and digested in a sterile filtered solution containing DMEM/F12 (Life technologies) with collagenase II (1 mg/ml) and 15 mg/ml fatty acid-free bovine serum albumin (BSA) (SigmaAldrich) and shaken gently for 20 min at 37°C. After the suspension had been filtered through a cell strainer (70 μm) it was left to settle for 5 min. A syringe with a needle (21 gauge, 0.8 mm × 50 mm) was used to remove the intermediate layer (underneath floating adipocytes) which was subsequently passed through a 30 μm filter. This cell suspension was centrifuged for 7 min at 800 g and re-suspended in DMEM/12, 1% penicillin/streptomycin, 10% fetal bovine serum (FBS) and seeded in a 10 cm dish and cultured at 37 °C in a humidified incubator with 5% CO_2_. Media was changed the day after isolation and then every other day until the cells reached 80% confluence, at which time they were frozen down in passage 0 (P0) in liquid nitrogen and stored at −180°C for up to 5 years.

### Cell culture and differentiation

Adipose-derived progenitors from non-obese (*n* = 13) and obese (*n* = 13) women were cultured in 60 mm culture plates with change of proliferation media (DMEM/F12, 10% FBS, 1% Penicillin-Streptomycin (P/S) (Invitrogen) and 1 nM Fibroblast growth factor-acidic (FGF-1) (ImmunoTools) every second day. Cells were harvested at 80% confluency for RNA isolation with the Trizol reagent. In a second batch of cells, adipocyte differentiation was induced 2 days after pre-adipocyte cultures were 100% confluent with media containing DMEM/F12, 1% P/S, 100 nM dexamethasone (Sigma-Aldrich), 100 nM insulin (Actrapid, Novo Nordisk), 1 μM rosiglitazone (Sigma-Aldrich), 500 μM isobutylmethylxanthine (Sigma-Aldrich), 2 nM triiodothyronine (T3) (Sigma-Aldrich), biotin 33 μM (Sigma-Aldrich), pantothenic acid 17 μM (Sigma-Aldrich) and 10 μg/ml transferrin (Sigma-Aldrich) which was changed on day 0, 3 and 6 of differentiation. Hereafter the differentiation medium was changed on day 9 and 12 with DMEM/F12 containing 1% Penicillin-Streptomycin, 10 nM dexamethasone (Sigma-Aldrich) and 10 nM insulin (Actrapid, Novo Nordisk) (Lee et al., 2012). On day 15, RNA was harvested in Trizol reagent. Furthermore, adipose-derived progenitors from non-obese women from visceral and subcutaneous adipose tissue were also differentiated in parallel stimulated with 10 ng/ml TNF (R&D systems) or saline control in the differentiation medium, at every media change. On day 15, RNA was harvested, and media collected.

### Flow cytometry staining

ASPCs were cultured in 10 cm dishes until they reached 90% confluence. Cells were released from the plate using Tryple (Invitrogen) and counted followed by a wash with PBS and heat inactivated FBS (FACS buffer). Cells were resuspended to a concentration of 1 × 10^^6^ cells/mL in FACS buffer and 50,000 cells were transferred to each well of a 96-well plate. Cells were stained for surface markers using the following antibodies: anti-human CD31-FITC (BD Biosciences Catalogue number: 557508), CD90-PerCP-Cy5.5 (BD Bioscience, Catalogue number: 561557), CD166-PE (BD Bioscience, Catalogue number: 559263) and CD56-BV605 (BD Bioscience, Catalogue number: 562779). Prior to the final experiment, a titration was performed to identify the optimal antibody dilution. Antibodies were combined as indicated in the figures with a final dilution of 1:50. 100 μl of the antibody mix (containing 2ul of each antibody diluted into FACS buffer) was added to each well and incubated for 20 min. Cells were washed and resuspended in wash buffer (PBS containing 2% heat inactivated FBS and 0.5 mM EDTA). A compensation plate was prepared with positive anti-mouse IgG beads together with individual antibodies for single staining including unstained cells and beads. Data was analyzed using FCS Express 7 Flow version 7.04.0014.

### Protein concentration in cell culture media

To evaluate the secretion of IL-6 and C-C motif chemokine 2 (encoded by *CCL2*), media was collected from cell cultures differentiated in the presence (*n* = 10) or absence of TNF (*n* = 10) and adiponectin in the presence (*n* = 13) or absence (*n* = 13) of TNF. On day 15 after six hours of incubation in F12/DMEM, media was analyzed with the Meso Scale Discovery technology (MSD) according to the manufacturer’s protocols. Samples and standards were measured in duplicates, and measurements with a coefficient of variation (CV) > 20% were excluded. Samples were excluded on this criterion for both measurement of IL-6 (*n* = 4) and adiponectin (*n* = 4).

### RNA isolation and qPCR

Adipose tissue samples from non-obese (*n* = 14) and women living with obesity (*n* = 14) were homogenized using steel beads (approximately 60 mg SAT and 60 mg VAT in 1 ml of Trizol (Invitrogen, United States) using a Tissuelyzer (Qiagen, Retsch GmbH, Germany). Total RNA was isolated from adipose tissue according to the manufacturer’s recommendations, with some adjustments including using 300 μl/ml chloroform per ml Trizol instead of 200 μl/ml and washed twice with ethanol (Invitrogen,CA, United States). Total RNA was extracted from cultured adipocyte progenitor cells and mature adipocytes with Trizol (Invitrogen, Carlsbad, CA, United States). Total RNA was dissolved in RNase-free water and quantified using a Nanodrop 1,000 spectrophotometer (Thermo Scientific, Wilmington, DE, United States). 500 ng RNA were reverse transcribed into cDNA using the High Capacity Reverse Transcription kit (Applied Biosystems, Foster City, CA, United States) according to the manufacturer’s protocol. qPCR was performed in duplicates with 10 μL reactions containing 3 μL cDNA in a 1:10 dilution, and either SYBR green master mix or TaqMan Universal PCR Master mix (Applied Biosystems, Branchburg, NJ, United States) using 384-well plates loaded onto a ViiA^TM^ 7 Sequence Detection system (Applied Biosystems, Foster City, CA, United States), with 40 cycles (two steps: 95°C for 15 s followed by 60°C for 30 s), and meltingcurves for SYBR q-PCR reactions to ensure single product formation. Standard dilution curves were carried out for each primer set. Primers were designed using the Primer-Blast (NCBI) software and ordered from TAG Copenhagen A/S. Primer sequences are listed in Table 2. All target genes were normalized to peptidylprolyl isomerase A (*PPIA*). Target mRNA was normalized to *PPIA* expression using the ΔΔCT method (User Bulletin No. 2, ABI PRISM 7700 Sequence Detection System). The relative expression ΔΔCT was calculated by relating ΔCT values for each sample to the average subcutaneous ΔCT value for the non-obese NGT patients.

**TABLE 2:** qPCR primer sequences.

Primer	Forward	Reverse	Probe
*TNF*	GGA​GAA​GGG​TGA​CCG​ACT​A	TGCCCAGACTCGGCAAAG	CGC​TGA​GAT​CAA​TCG​GCC​CGA​CTA
*IL6*	CTG​CAG​AAA​AAG​GCA​AAG​AAT​CTA​G	TCT​GTG​CCT​GCA​GCT​TCG​T	CACCCCTGACCCAAC
*CCL2*	CCC​CAG​TCA​CCT​GCT​GTT​AT	AGA​TCT​CCT​TGG​CCA​CAA​TG	
*FABP4*	CCT​TTA​AAA​ATA​CTG​AGA​TTT​CCT​TCA	GGA​CAC​CCC​CAT​CTA​AGG​TT	
*PPARG*	GAAACTTCAAGAGTACCA AAGTGCAA	AGG​CTT​ATT​GTA​GAG​CTG​AGT​CTT​CTC	CAA​AGT​GGA​GCC​TGC​ATC​TCC​ACC​TTA​TT
*LPL*	CCA​TGG​CTG​GAC​GGT​AAC​AGG​A	GCC​CGC​GGA​CAC​TGG​TAA​T	
*PPIA*	ACG​CCA​CCG​CCG​AGG​AAA​AC	TGC​AAA​CAG​CTC​AAA​GGA​GAC​GC	
*ADIPOQ*	AAA​ACC​TCC​CCC​AAG​CAG​AGC​TTC	TGA​GGA​ACA​GGG​ATG​AGT​TCA​GCA	
*BST2*	TGA​TGG​CCC​TAA​TGG​CTT​CC	TTC​TCT​TCT​CAG​TCG​CTC​CAC	
*TP53*	AGG​CCT​TGG​AAC​TCA​AGG​AT	CCC​TTT​TTG​GAC​TTC​AGG​TG	

### qPCR array analysis

Custom designed StellAR-ray qPCR arrays (384-well plate with 4 × 96 genes) (Lonza, Basel, Switzerland) were utilized according to the manufacturer’s instruction to measure gene-expression in paired subcutaneous and visceral adipose-derived progenitors (from *n* = 13 non-obese and *n* = 13 obese). The qPCR-array was performed with 10 μL in single reactions containing 2 μL cDNA (from the above-described cDNA solution)*,* 3 μL water and 5 μL SYBR green master mix, added to each well of the StellARray 384-well plate (four samples for one plate). The 384-well plates were loaded onto a ViiA^TM^ 7 Sequence Detection system (Applied Biosystems, Foster City, CA) and followed the thermal program: 2 min at 50°C, (30 s at 95°C) and 40 cycles (two steps: 95°C for 15 s followed by 60°C for 1 min) and melting curves for SYBR q-PCR reactions to ensure single product formation. The global data set was analyzed using GenEx version 6. Data was normalized to the average expression of PPIA, ACTN and 18S and then log transformed to reach linearity. The list of all analyzed genes, plate set-up and ct-values is included in (Supplementary Material S1).

### Statistics

All analyses were performed using the Graphpad Prism 8 software. Subject characteristics were analyzed with student’s t-tests. Two-way Anova analyses, or Mixed models when samples were missing, were used to assess differences between adipose depot (subcutaneous and visceral) origin and obesity (non-obese and obese) or TNF treatment (TNF and control) and whether there was any interaction between these factors. Post-hoc tests were performed when relevant. A *p*-value of <0.05 was considered significant.

## Results

### Human subjects

We included morbidly obese women undergoing gastric bypass surgery (*n* = 14; BMI>35 kg/m^2^), compared to non-obese women undergoing cholecystectomy (*n* = 14; BMI<30 kg/m^2^) (Table 1). The surgery allowed for collection of visceral and subcutaneous adipose tissue (VAT and SAT, respectively) from each of the subjects. All subjects were normal glucose tolerant, but the obese women had higher HOMA-IR compared to the non-obese women (3.46 ± 0.63 vs. 1.65 ± 0.25; *p* < 0.05) (Table 1).

### Differential regulation of inflammatory signature in visceral versus subcutaneous adipose tissue in obesity.

**FIGURE 1:**
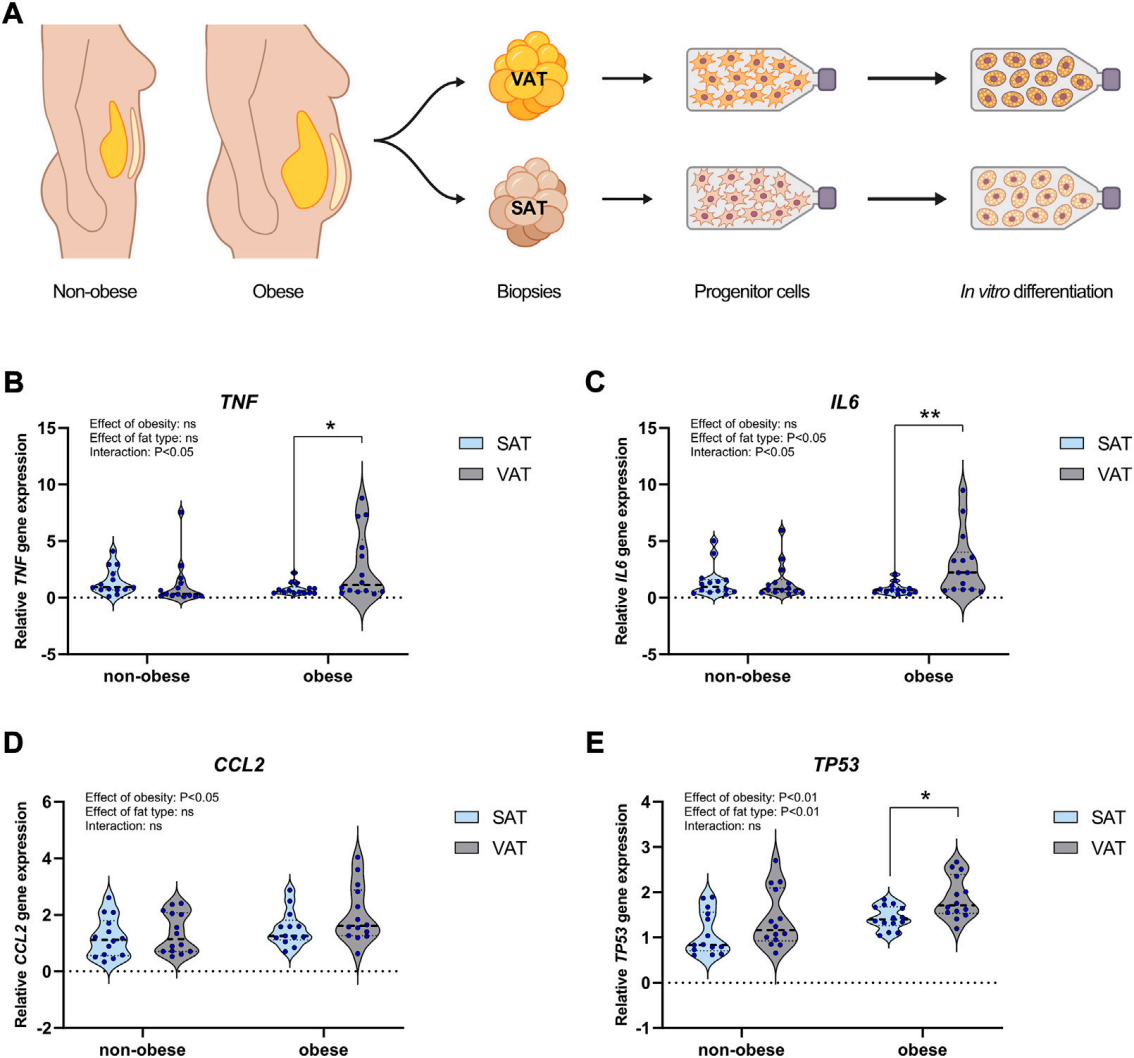
Gene expression of immunogenic markers in adipose tissue of non-obese women and women living with obesity. Adipose tissue biopsies from subcutaneous and visceral adipose regions were collected from gallstone patients (non-obese, *n* = 14) and gastric surgery patients (obese, *n* = 14). **(A)** Cartoon of adipose tissue depots and isolated cells. **(B–E)** qPCR analysis of the immunogenic markers *TNF*, *IL6*, *CCL2* and *TP53* in paired samples of subcutaneous adipose tissue (SAT) and visceral adipose tissue (VAT) obtained from non-obese women and women living with obesity. Differences between groups and adipose depots were assessed using two-way anova with subsequent post-tests. Data are presented as violin plots with thick dotted line showing median and thin dotted lines showing quartiles. Two-way anova assessed difference between groups and depots. Results from significant post-tests are shown as: *p < 0.05, **p < 0.01, ***p < 0.001, ****p < 0.0001.

To investigate the differences between ASPCs derived from abdominal VAT and SAT of women living with obesity (*n* = 14) and non-obese women (*n* = 14), we collected surgical biopsies and analyzed marker gene expression in tissue, ASPCs and *in vitro* differentiated adipocytes (Figure 1A). We first isolated RNA and measured the expression of inflammatory markers in tissue biopsies from the VAT and SAT depots of the women living with obesity (*n* = 14) and non-obese women (*n* = 14). We found that the pro-inflammatory cytokines *TNF* and *IL6* were higher expressed in visceral compared to subcutaneous adipose tissue of subjects living with obesity, whereas no depot-dependent difference was observed in non-obese women (Figures 1B,C). In contrast, *CCL2* was not different between VAT and SAT, but displayed an overall elevation in samples from subjects living with obesity (Figure 1D). Finally, *TP53* was regulated both between depots and in obesity, with a higher expression in VAT compared to SAT in the obesity condition (Figure 1E). *TP53* is primarily known as a tumour-suppressor protein, but is also described as a regulator of metabolism and adipose tissue inflammation (Shimizu et al., 2012). Our data thus indicated that women living with morbid obesity mainly have increased inflammation of their VAT, whereas their SAT appears less severely affected based on the markers measured.

### Adipogenic and immunogenic signatures in visceral versus subcutaneous adipocytes

**FIGURE 2:**
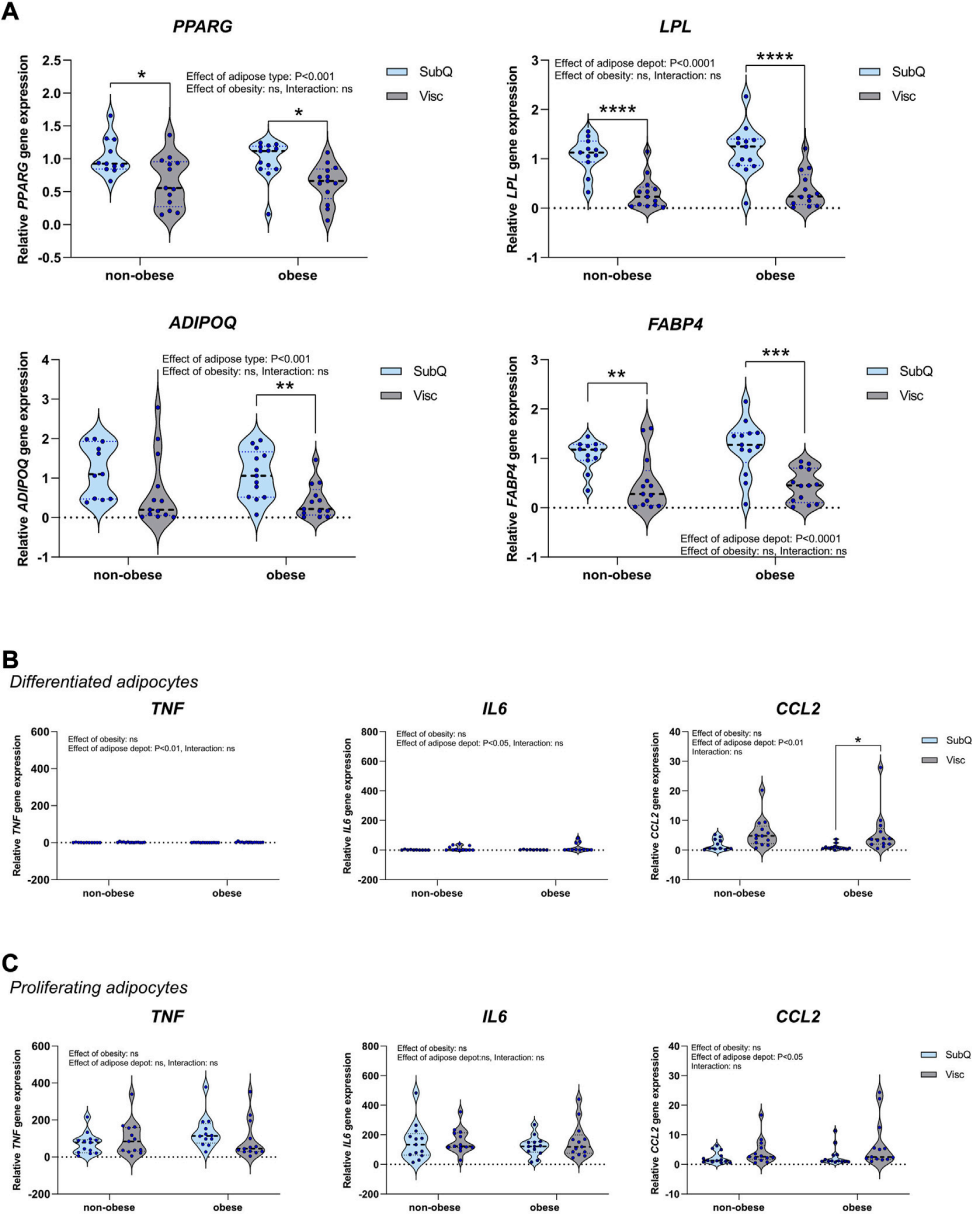
Adipogenic and immunogenic gene expression in APSCs from SAT and VAT of non-obese women and women living with obesity. ASPCs were isolated from the subcutaneous and visceral adipose biopsies from women living with obesity (*n* = 13) and non-obese women (*n* = 13). **(A)** qPCR analysis was used to measure the relative expression of the adipogenic markers *PPARG*, *LPL*, *ADIPOQ* and *FABP4*. QPCR analysis was used to measure the relative expression of the immunogenic markers *TNF*, *IL6* and *CCL2* in **(B)** Differentiated adipocytes and **(C)** Proliferating APSCs. Data are presented as violin plots with thick dotted line showing median and thin dotted lines showing quartiles. Two-way anova assessed difference between groups and depots. Results from significant post-tests are shown as: *p < 0.05, **p < 0.01, ***p < 0.001, ****p < 0.0001.

We next compared the gene expression of adipogenic and immunogenic markers in ASPCs derived from VAT and SAT of women living with obesity (*n* = 13) and non-obese women (*n* = 13). The ASPCs were isolated from adipose tissue biopsies at the day of surgery, expanded and frozen in liquid nitrogen. When the full cohort was established, cells were differentiated *in vitro* in parallel using the same differentiation protocol, into lipid-droplet containing adipocytes. Previous studies have reported on less adipogenic potential in human visceral compared to subcutaneous adipocytes (Macotela et al., 2012), although biological variation might likely also play a role. We therefore measured adipogenic markers in our sample-set of *in vitro* differentiated ASPCs derived from 26 different individuals. We found that the adipogenic markers *PPARG*, *LPL, ADIPOQ*, *FABP4* were indeed lower expressed in the visceral adipocytes compared to the subcutaneous (Figure 2A). Adipocytes have been reported with an ability to acquire immunogenic properties (Caputa et al., 2022). To assess potential differences between our groups, we therefore next measured a subset of immunogenic markers in the adipocytes. Where *CCL2* was higher expressed in the visceral adipocytes, *TNF* and *IL6* were extremely low expressed, closed to undetermined (Figure 2B). We then measured the same markers in proliferating cells and related the expression to the differentiated control cells for comparison. TNF and IL6 were substantially higher expressed in the proliferating cells compared to differentiated, but only CCL2 was higher expressed in visceral compared to subcutaneous cells (Figure 2C). To address whether insulin resistance could affect the adipogenic capacity of ASPCs, we reorganized the cell groups after HOMA-IR, so that subjects with HOMA-IR >2 were in the HOMA high group, and the rest in the HOMA low group (Supplementary Figures S1A–C). Because insulin and glucose data were not available for all subjects, this resulted in slightly lower n-numbers for each group, as specified in the figure legend. Interestingly, we found no obesity-dependent and no HOMA-IR dependent effects on either adipogenic or immunogenic markers, raising the possibility that ASPCs remain functional in a progenitor state despite exposure to an obesity-dependent microenvironment.

### BST2 is a marker of visceral adipose stem and progenitor cells

**FIGURE 3:**
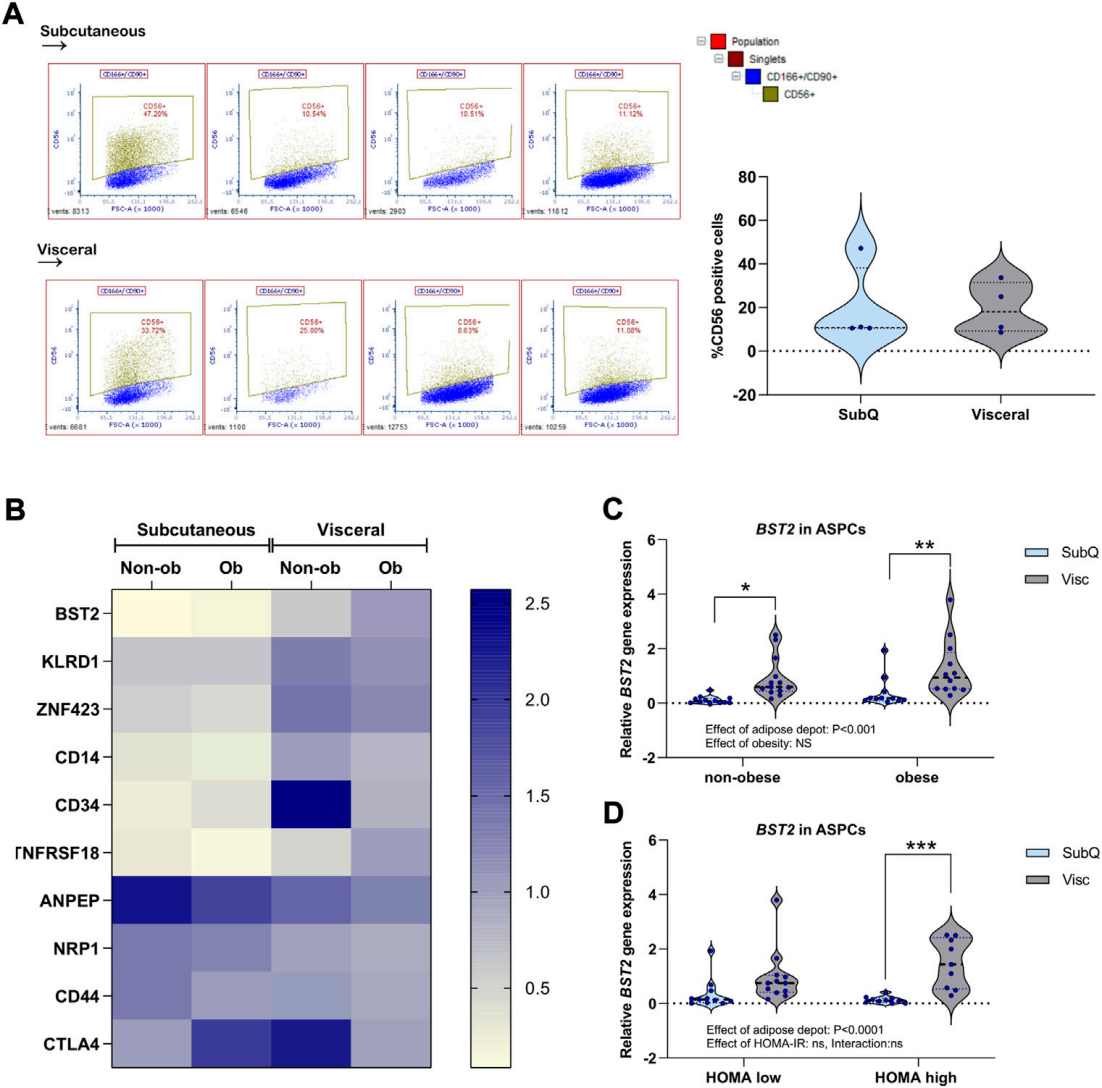
Characterization of APSCs derived from human SAT and VAT. **(A)** APSCs derived from SAT and VAT were cultured and characterized for surface expression of CD90, CD166 and CD56 using flow cytometry. Granularity and relative size were evaluated by side- and forward scatter area (SSC-A and FSC-A) to select the preadipocyte population for analysis. From these cells, single cells were selected by evaluation of forward scatter height and width (FSC-h and FSC-W). Within this population, cells positive for CD166, CD90 and CD55 were selected. Gating strategy: preadipocytes/singlets/CD166+CD90+/CD56+. Flow cytometry illustrated a smeared DIM CD56^+^ population within the preadipocyte population in both visceral and subcutaneous adipocytes. Right: summary graph of CD56^+^ APSCs (*n* = 3) **(B)** Sub-cultured APSCs harvested at 80% confluency was analyzed using GenEx qPCR array. Heatmap shows top 10 genes with lowest *p*-values from pairwise comparisons among the four groups. All data is shown in (Supplementary Material S1) **(C)** Data derived from the qPCR array on APSCs showing *BST2* mRNA levels. **(D)** Data derived from the qPCR array on APSCs showing *BST2* mRNA levels when samples were reorganized based on HOMA-IR. As clinical data were not available for all subjects, the sample groups were slightly reduced compared to the non-obese vs obese groups. HOMA high represents HOMA-IR values over 2 and HOMA low represents HOMA-IR values under or equal to 2. SAT HOMA low: *n* = 11 (7 non-obese, 4 obese); SAT HOMA high: *n* = 10 (4 non-obese, 6 obese); VAT HOMA low: *n* = 12 (8 non-obese, 4 obese); VAT HOMA high: *n* = 9 (4 non-obese, 6 obese). Data are presented as violin plots with thick dotted line showing median and thin dotted lines showing quartiles. Two-way anova assessed difference between groups and depots. Significant post-test is shown as: *p < 0.05, **p < 0.01, ***p < 0.001, ****p < 0.0001.

We next aimed to further address how proliferating APSCs were affected by obesity in the two adipose depots from the same group of women living with obesity (*n* = 13) and non-obese women (*n* = 13) as described above. Adipose progenitors that we isolated from the stromal vascular fraction of the tissue biopsies were sub-cultured and cells were harvested at 90% confluence (passage 3–4) and characterized using flow cytometry. Cells were negative for the endothelial marker CD31 whereas the entire cell populations (debris and doublets excluded) of both visceral and subcutaneous origin, were positive for CD90 and CD166, previously described to predict cells with high adipogenic potential (Cawthorn et al., 2012). This data suggested equal purity of the isolated visceral and subcutaneous progenitors. To further characterize and address the higher expression of immunogenic markers in the visceral compared to subcutaneous adipocytes, we analysed the pure population for CD56, also known as Neural cell adhesion molecule 1 (NCAM1). CD56 is a present on the surface of several immune cells including NK cells, T-cells and dendritic cells (De et al., 2017), but is also present in mesenchymal stem cell populations (https://www.proteinatlas.org/), (Thul et al., 2017). We observed that around 20% of the cells were positive for CD56 in our cultures. However, we observed no difference in CD56 positivity between visceral and subcutaneous APSCs (Figure 3A). To further address the immunogenic profile of our cohort of APSCs, we next designed a PCR array consisting of 82 genes. These genes included multiple immune cell markers and immune related genes. We measured the gene expression of these markers in APSCs derived from women living with obesity (*n* = 11) and non-obese women (*n* = 13) (Figure 3B; Supplementary Material S1). The analysis was performed on sub-cultured APSCs harvested at 80% confluence. Interestingly, we observed a substantially higher expression of *CD34* in the progenitors derived from the visceral depot compared to subcutaneous progenitors from non-obese subjects (Figure 3B; Supplementary Material S1). CD34 was recently reported as a marker for adipocytes with high rates of lipid flux (Raajendiran et al., 2019). The array also identified *BST2* as a visceral marker gene (Figures 3B,C; Supplementary Material S1)*. BST2* was higher expressed in the visceral depot in APSCs from both non-obese women and women living with obesity. Interestingly, the difference was more pronounced in obesity and even more so in insulin resistance (Figures 3C,D). *BST2* encodes an antiviral factor, Bone marrow stromal antigen 2 (BST2, also known as Tetherin) (Perez-Caballero et al., 2009), further emphasizing the immunogenic phenotype of visceral adipocytes.

### A TNF microenvironment favours an immunogenic signature in adipose stem and progenitor cells from both depots

**FIGURE 4:**
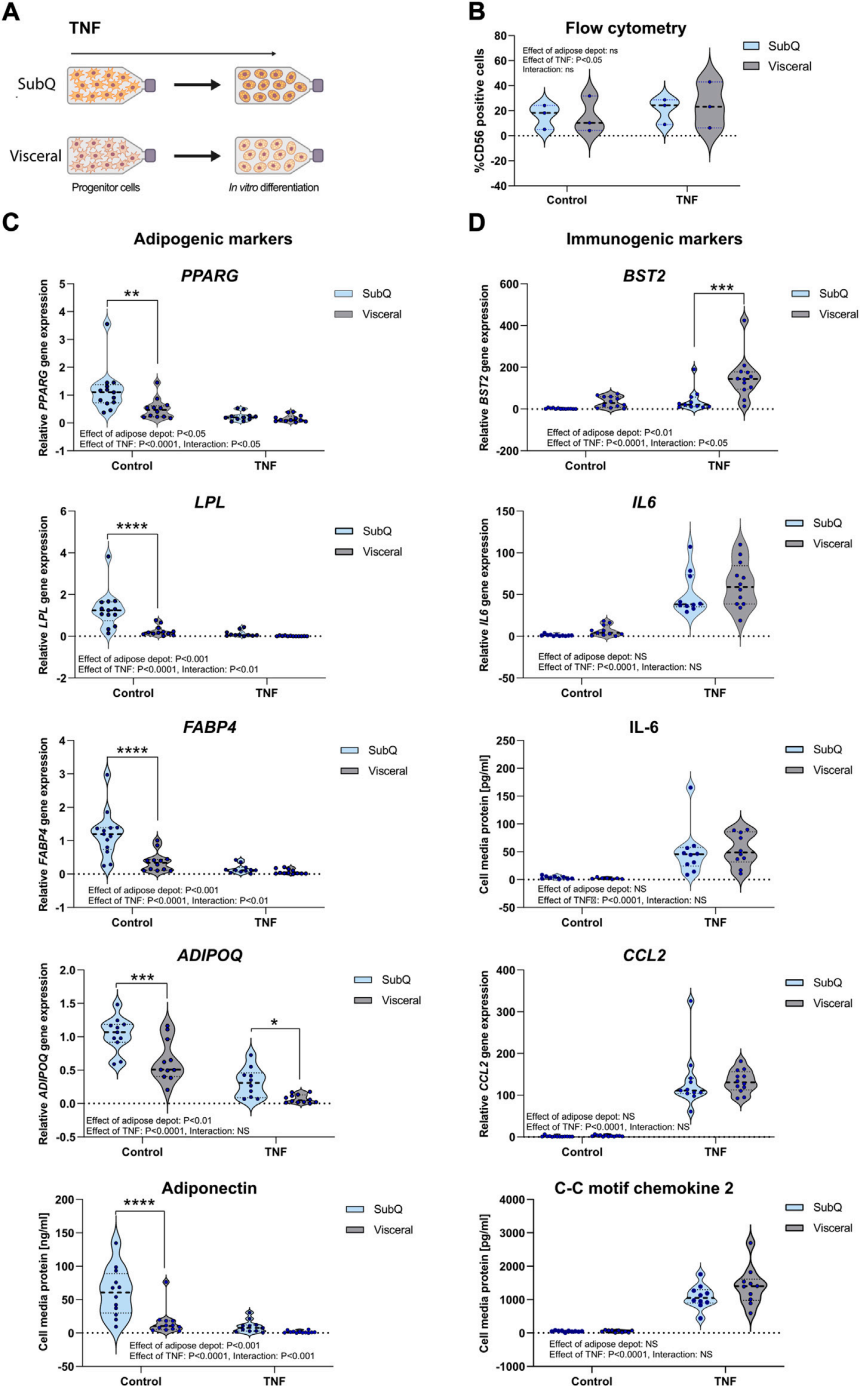
Adipogenic and immunogenic gene expression in APSCs stimulated with TNF during differentiation. APSCs derived from SAT and VAT of non-obese women (*n* = 13) were differentiated in media supplemented with TNF or saline control. **(A)** Cartoon showing experimental set-up. **(B)** Flow cytometry measuring CD56^+^ positive cells in APSCs exposed to 10 ng/ml TNF or saline control for 48 h (*n* = 3/condition). **(C)** qPCR analysis was used to measure the relative expression of the adipogenic markers *PPARG*, *LPL*, *FABP4* and *ADIPOQ*. Adiponectin protein levels was measured in the cell media using ELISA. **(D)** qPCR analysis was used to measure the relative expression of the immunogenic markers *BST2*, *IL6* and *CCL2.* IL-6 and C-C motif chemokine-2 protein levels were measured in the cell media using ELISA. Data are presented as violin plots with thick dotted line showing median and thin dotted lines showing quartiles. Two-way anova assessed difference between groups and depots. Results from significant post-tests are shown as: *p < 0.05, **p < 0.01, ***p < 0.001, ****p < 0.0001.

As the APSCs derived from women living with obesity were not different in terms of adipogenic signatures, we hypothesized that the microenvironment during adipogenesis played a greater role than the exposure to an “obesity-microenvironment” at progenitor state. We were specifically interested in comparing the different responses between APSCs derived from the visceral and the subcutaneous depots and to address whether an inflammatory microenvironment could reprogram the subcutaneous adipocyte progenitor cells and make them more “visceral-like” (Figure 4A). First, we reassessed the flow cytometry analysis in *n* = 3 cell cultures from each depot, using the same strategy as in Figure 3A. Interestingly, following 48 h of TNF, the proportion of CD56 positive cells modestly increased, but with no difference between the visceral and subcutaneous APSCs (Figure 4B). We next differentiated subcutaneous and visceral APSCs derived from the full sample set of non-obese women (*n* = 13, in the presence of recombinant TNF (10 ng/ml) or saline control, for 15 days. This chronic TNF treatment during ASPCs differentiation hampered adipogenic capacity in terms of reduced gene expression of *LPL*, *FABP4 and PPARG* (Figure 4C). Strikingly, differentiation with TNF reduced the difference in gene expression of these adipogenic genes between the visceral and subcutaneous adipocytes. Interestingly, *ADIPOQ* was differentially expressed between subcutaneous and visceral adipocytes both in the control state and in the TNF treated cells, highlighting this gene as a major intrinsic marker of subcutaneous identity. In contrast, the protein levels of adiponectin in the cell media followed the same regulation pattern as the other adipogenic markers (Figure 4C), i.e., the difference in adipogenic capacity between visceral and subcutaneous adipocytes observed under normal conditions is minimized when cells are exposed to a TNF-enriched microenvironment during differentiation. Conversely, the immunogenic markers were upregulated following TNF exposure during the differentiation (Figure 4D). The treatment erased all differences in immunogenic profile between depots except for *BST2*, where a difference between adipose depots was more pronounced in the TNF-stimulated cells, underscoring its immunogenic role.

## Discussion

We studied visceral and subcutaneous APSCs derived from non-obese women and from women living with obesity. We found that the adipogenic and immunogenic signatures of visceral APSCs were differential to the signatures of subcutaneous APSCs independent of obesity, despite obesity-related higher expression of inflammatory markers in the visceral adipose tissue. Adding the pro-inflammatory cytokine TNF during APSC differentiation diminished the difference between visceral and subcutaneous adipocytes in terms of adipogenic and immunogenic signatures, emphasizing the importance of the microenvironment during APSC differentiation in determining the destiny of the mature adipocyte. Recent studies at single cell resolution provides further insight in human adipose depot-dependent differences and adipocyte function, raising the idea that the immunogenic and metabolic properties of the adipose tissue is dependent on the composition and location of different adipocyte subtypes (Scheele and Wolfrum, 2021; Scheele and Nielsen, 2022). Adipocytes interact with adipose-resident macrophages during the development of obesity-induced insulin resistance (Weisberg et al., 2003; Xu et al., 2003). In this respect, our tissue biopsy data likely reflects elevated low-grade inflammation, primarily occurring in the visceral adipose depot. However, recent studies are bringing a new perspective on the interactions between macrophages and APSCs and on the immunogenic capacity of adipocytes. For example, using single cell spatial transcriptomics, a subtype of APSCs was found to associate with macrophages (Bäckdahl et al., 2021) and more recently it was described that at least rodent adipocytes can be rewired from lipid metabolism to acquiring an immunogenic phenotype and participate in host defence in response to infectious stimuli (Caputa et al., 2022). These latter findings are consistent with our data on the intrinsic signatures and inverse relationship between immunogenic and adipogenic signatures of visceral and subcutaneous adipocytes. These observations further raise the idea that adipocytes, when triggered, actively contribute to the low-grade inflammation produced in association with insulin resistance. Indeed, TNF stimulation of *in vitro* adipocytes have previously been shown to increase the production of inflammatory cytokines (Wang and Trayhurn, 2006), and hamper the adipogenic program (Ruan et al., 2002; Cawthorn et al., 2007; Hammarstedt et al., 2007; Cawthorn and Sethi, 2008). In the current study, we observed that a switch from an adipogenic signature to an immunogenic signature in response to TNF occurred in both visceral and subcutaneous adipocytes. However, some intrinsic differences remained. For example, we identified the antiviral factor, *BST2*, as selectively expressed in visceral APSCs and adipocytes. *BST2* was further increased by chronic TNF treatment during differentiation, in particular in the visceral adipocytes. These intrinsic differences could be important for our understanding of the association between excessive accumulation of visceral adipose tissue in the abdominal region and metabolic disease.

A limitation with our study is that we focus on adipose tissue from women and the relation between APSCs from SAT and VAT might be different in men. Furthermore, we have only measured marker genes (mRNA and secreted proteins), rather than studying function. However, the adipogenic and immunogenic markers measured here are well described, and our data therefore still provides new insights. A strength with our study is that we have included cell cultures derived from 26 human subjects with paired SAT and VAT biopsies. Thus, the differences we observe are likely not due to technical variation but represent biological differences between SAT and VAT derived APSCs. In conclusion, our data underscore the intrinsic differences between human visceral and subcutaneous adipocytes, suggest that dysregulation of adipocytes in obesity occurs at a post-progenitor stage, and highlight an inflammatory microenvironment as a major constraint of human adipogenesis.

## Data Availability

The original contributions presented in the study are included in the article/Supplementary Material, further inquiries can be directed to the corresponding authors.
