# Supplementary material for: Human visceral and subcutaneous adipose stem and progenitor cells retain depot-specific adipogenic properties during obesity
Source: Front Cell Dev Biol. 2022 Oct 17;10:983899. doi: 10.3389/fcell.2022.983899 (PMC9629396; doi:10.3389/fcell.2022.983899)
Supplement: Supplementary file 2 [file DataSheet2.docx]

**Supplementary figure legend**

**Figure S1. Adipogenic and immunogenic gene expression in APSCs from SAT and VAT of non-obese women and women living with obesity**

ASPCs were isolated from the subcutaneous and visceral adipose biopsies from women living with obesity (n=13) and non-obese women (n=13). Samples were reorganized based on HOMA-IR. As clinical data were not available for all subjects, the sample groups were slightly reduced compared to the non-obese vs obese groups. HOMA high represents HOMA-IR values over 2 and HOMA low represents HOMA-IR values under or equal to 2. SAT HOMA low: n=11 (7 non-obese, 4 obese); SAT HOMA high: n=10 (4 non-obese, 6 obese); VAT HOMA low: n=12 (8 non-obese, 4 obese); VAT HOMA high: n=9 (4 non-obese, 6 obese).  **(A)** qPCR analysis was used to measure the relative expression of the adipogenic markers *PPARG*, *LPL*, *ADIPOQ* and *FABP4*. QPCR analysis was used to measure the relative expression of the immunogenic markers *TNF*, *IL6* and *CCL2* in **(B)** Differentiated adipocytes and **(C)** Proliferating APSCs. Data are presented as violin plots with thick dotted line showing median and thin dotted lines showing quartiles. Two-way anova assessed difference between groups and depots. Results from significant post-tests are shown as: *P<0.05, **P<0.01, ***P<0.001, ****P<0.0001
